# Decline to near-zero malaria hospitalisation over 35 years on the Kenyan Coast

**DOI:** 10.1186/s12936-026-06081-x

**Published:** 2026-08-07

**Authors:** Alice Kamau, Moses M. Musau, Neema Mturi, Shebe Mohammed, Gabriel Mwambingu, David Walumbe, Amek Nyaguara, Thomas N. Williams, Philip Bejon, Robert W. Snow

**Affiliations:** 1 KEMRI-Wellcome Trust Research Programme, Nairobi, Kenya; 2 KEMRI Centre for Global Health Research, Kisumu, Kenya; 3 Department of Vector Biology, Liverpool School of Tropical Medicine, Liverpool, UK; 4 KEMRI-Wellcome Trust Research Programme, Kilifi, Kenya; 5 Faculty of Medicine, Imperial College, St Mary’s Hospital, London, UK; 6 Centre for Tropical Medicine and Global Health, Nuffield Department of Medicine, University of Oxford, Oxford, UK; 7 Modernising Medical Microbiology, Nuffield Department of Medicine, University of Oxford, Oxford, UK

**Keywords:** Malaria, Hospitalisation, Age, Severe malaria, Trends, Africa

## Abstract

**Background:**

Few longitudinal studies have examined the long-term health impact of changing malaria prevention and treatment strategies in Africa. This study analyses 35 years of clinical surveillance among paediatric malaria admissions in Kilifi, Kenya.

**Methods:**

Children aged 1 month to 14 years who were residents of rural Kilifi Health and Demographic Surveillance System and admitted to Kilifi County Hospital between January 1990 and December 2024 were included. Community malaria exposure was estimated using infection prevalence among children admitted for trauma, elective surgery, bites and neoplasms. Malaria admissions were defined as hospitalisations with a positive blood slide and a primary or co-morbid malaria diagnosis. Severe malaria phenotypes including severe anaemia, cerebral malaria, hyper-parasitaemia and in-hospital mortality were also examined. Binomial and Poisson regression models assessed temporal differences, using 1990–1996 as the reference period.

**Results:**

Community malaria prevalence declined from 35% (95% CI 31, 39) in the 1990s to 2% (95% CI 1, 4; p < 0.001) in 2020–2024. Malaria hospitalisations declined from a peak of 25.5 per 1,000 children per annum (p.a.) (95% CI 24.4–26.6) in 1999 to 0.65 (95% CI 0.59–0.72; p < 0.001) between 2020–2024. The median age of malaria increased from 19 months (IQR: 12, 39) between 1990–1996 to a peak of 48 months (IQR: 28, 78) between 2012–2019. Cerebral malaria became proportionally more common than severe anaemia over time. Malaria-specific hospitalised mortality rates declined from 0.43 per 1,000 children p.a. (95% CI 0.38–0.49) in the 1990s to 0.03 (95% CI 0.02–0.05; p < 0.001) during the period 2020–2024.

**Conclusions:**

Hospital admission with malaria among children in Kilifi is now uncommon. Sustained reductions in parasite exposure have altered the clinical profile of disease presentation, including both phenotype and age distribution, without resulting in a cumulative increase in disease burden. Expanded and sustained coverage of effective long-lasting insecticidal nets, together with improved access to effective treatment, have likely contributed to this epidemiological transition.

**Supplementary Information:**

The online version contains supplementary material available at https://doi.org/10.1186/s12936-026-06081-x.

## Background

The Roll Back Malaria (RBM) initiative was launched in 1998 amid the alarming rise in malaria cases globally [1]. The African Heads of State signed a declaration in April 2000 committing their governments to reverse the malaria trend [2]. A new financing mechanism, the Global Fund to fight AIDS, TB and Malaria, was established in 2002 to increase and consolidate overseas development assistance (ODA) to tackle these three major causes of death, with a primary focus on Africa. In addition, the U.S. President’s Malaria Initiative (PMI) has played a pivot role in supporting malaria control across Africa [3]. Together, these funding mechanisms have provided an unprecedented, transformative grant-based financing, enabling African countries to scale up malaria control interventions.

Despite billions of dollars in ODA invested in malaria control, most Africa countries have limited direct empirical evidence of changes in disease burden. Infrequent national household surveys are not designed to measure disease incidence or provide reliable sub-national estimates of infection prevalence. Civil registration systems remain weak, and malaria specific mortality is difficult to measure in the absence of pre-mortem parasitological and clinical information [4]. As a result, the global community has relied on combinations of spatial and mechanistic models to predict changes in infection prevalence, morbidity and mortality, often built on sparse data and multiple assumptions [5]. However, models cannot substitute local surveillance and contextual information [6].

Hospital admissions provide a valuable source for empirical disease burden estimation [7]. Severe disease events presenting to emergency care facilities serve as a proxy for life-threatening events in the community that could be averted through timely treatment and effective interventions. Long-term hospital-based data of severe malaria is rare in Africa, with only two sites maintaining continuous surveillance since the launch of RBM, Manhica (Mozambique) [8] and Kilifi (Kenya) [9–11].

This study presents a comprehensive analysis of 35 years of paediatric malaria admissions in a rural population in Kilifi County, representing the longest continuous surveillance of hospitalised malaria in Africa. Temporal changes in disease burden and clinical epidemiology are examined in the context of evolving antimalarial treatment and vector control strategies, providing a perspective on how intervention strategies have shaped changing patterns of malaria risk and insights for future policy.

## Methods

### Study site

The study area is located along the Kenyan coast, within Kilifi County (Additional file 1). The KEMRI-Wellcome Trust Research programme was established in 1989, initiating a series of studies, including longitudinal clinical surveillance of severe malaria at Kilifi County Hospital (KCH) and a community-based randomised controlled trial of insecticide-treated bed net (ITN) between 1993 and 1995 [12]. In April 2002, demographic surveillance was established covering an area of 891 km^2^, extending 39 km north and 32 km south of Kilifi creek (Additional file 1). This area is referred to as the Kilifi Health and Demographic Surveillance System (KHDSS) [13]. The study population is served by KCH, where inpatient  care is provided free of charge through a partnership with the KEMRI-Wellcome Trust Research Programme. KCH is the primary referral hospital for severe illness in the study area, and there are no other major hospitals providing comprehensive inpatient care for severe disease. The KHDSS population is  re-enumerated on a rolling basis every 4 months to capture migration, pregnancies and vital events, alongside re-mapping of newly established homesteads [13]. The urban area immediately surrounding the hospital was excluded because of a high proportion of transient populations from other regions of Kenya (Additional file 1).

### Paediatric ward surveillance

In May 1989, the inpatient surveillance system was established using a standard paediatric admission record form that was completed by physicians to record age, gender, address, illness history, clinical investigations and laboratory results. Surveillance is conducted continuously 24/7 for all admissions to the paediatric ward and high-dependency unit. Malaria microscopy was performed on all admissions using 3% Giemsa-stained thick and thin blood films and parasite densities were computed based on the red or white blood cell counts. Full blood counts, including haemoglobin, were obtained using automated haematological analysers supplied by Beckman Coulter. Level of consciousness was assessed using the Blantyre Coma Score (BCS). Final diagnoses were assigned by the attending clinical team at discharge or death after reviewing all available clinical, laboratory results including blood and cerebrospinal fluid cultures, haematology, and radiological findings, together with the patient's response to treatment. Diagnoses were coded according to ICD-9 and subsequently ICD-10 classifications. The clinical team followed standard hospital clinical guidelines and diagnostic criteria throughout the surveillance period. Although individual clinicians changed over time, the surveillance procedures, diagnostic protocols, and coding practices remained consistent. Up to two diagnoses were recorded and, when both completed, they were treated as primary and secondary or co-primary, co-morbid conditions. Clinical and treatment guidelines for paediatric admissions have been consistent with changes in national and WHO policy recommendations. For the present analysis, all admissions aged 1 month to 14 years of age who were residents of the rural KHDSS were included from January 1990 to December 2024.

### Outcomes

Malaria admissions were defined as hospitalisations with a positive malaria blood slide and a discharge diagnosis of malaria recorded as primary or co-morbid. Among malaria admissions, severe malaria phenotypes with complete data since 1990 were defined as follows: (a) severe malaria anaemia (SMA), haemoglobin of < 5gm/dl; (b) cerebral malaria (CM), BCS < 3; (c) hyper-parasitaemia, parasite density > 250,000/μl; and (d) death during admission. These phenotypes may overlap, and analyses were based on events rather than individual children. Other WHO defined severe malaria manifestation [14], including respiratory distress, hypoglycaemia, prostration, multiple seizures and acute kidney injury were not systematically documented over the surveillance period and were therefore excluded.

### Statistical analysis

#### Community parasite prevalence

Community malaria exposure within the rural KHDSS was estimated from infection prevalence among children admitted for trauma (road traffic accidents, fractures, burns, accidental poisoning with insecticides or kerosene), elective surgery (e.g. circumcision repair, rectal prolapse, dental and hernia repair), snake and animal bites, and neoplasms [9–11]. Annual parasite prevalence was age-standardised to the 2–10 year age group [15], with confidence intervals estimated using 1,000 bootstrap iterations.

#### Annualised hospital admission incidence

Population denominators for children aged 0–14 years were obtained from rural KHDSS census enumerations conducted between 2003 and 2024. Populations for 1990–2002 were back-calculated using an annual population growth rate of 2.97% [13]. During periods affected by healthcare worker strikes, 2016–17 and 2021, we adjusted the annual population denominator proportionally to the duration of the strike to reflect the reduced period during which hospital admissions were not recorded. The age structure of annual populations was estimated using observed age proportions from combined census data since 2003, with the first year of life adjusted by 11/12.

Incidence was defined as minimum community-acquired hospital events per 1000 resident children per annum (p.a.), acknowledging that some events may not reach hospital. Annual incidence rates of malaria admissions were estimated using Poisson regression model, with malaria case counts as the dependent variable, year as the independent variable and population as an offset. Similar models were fitted for severe malaria phenotypes (CM, SMA, hyper-parasitaemia and malaria related deaths).

#### Context milestone periods

**Fig. 1:**
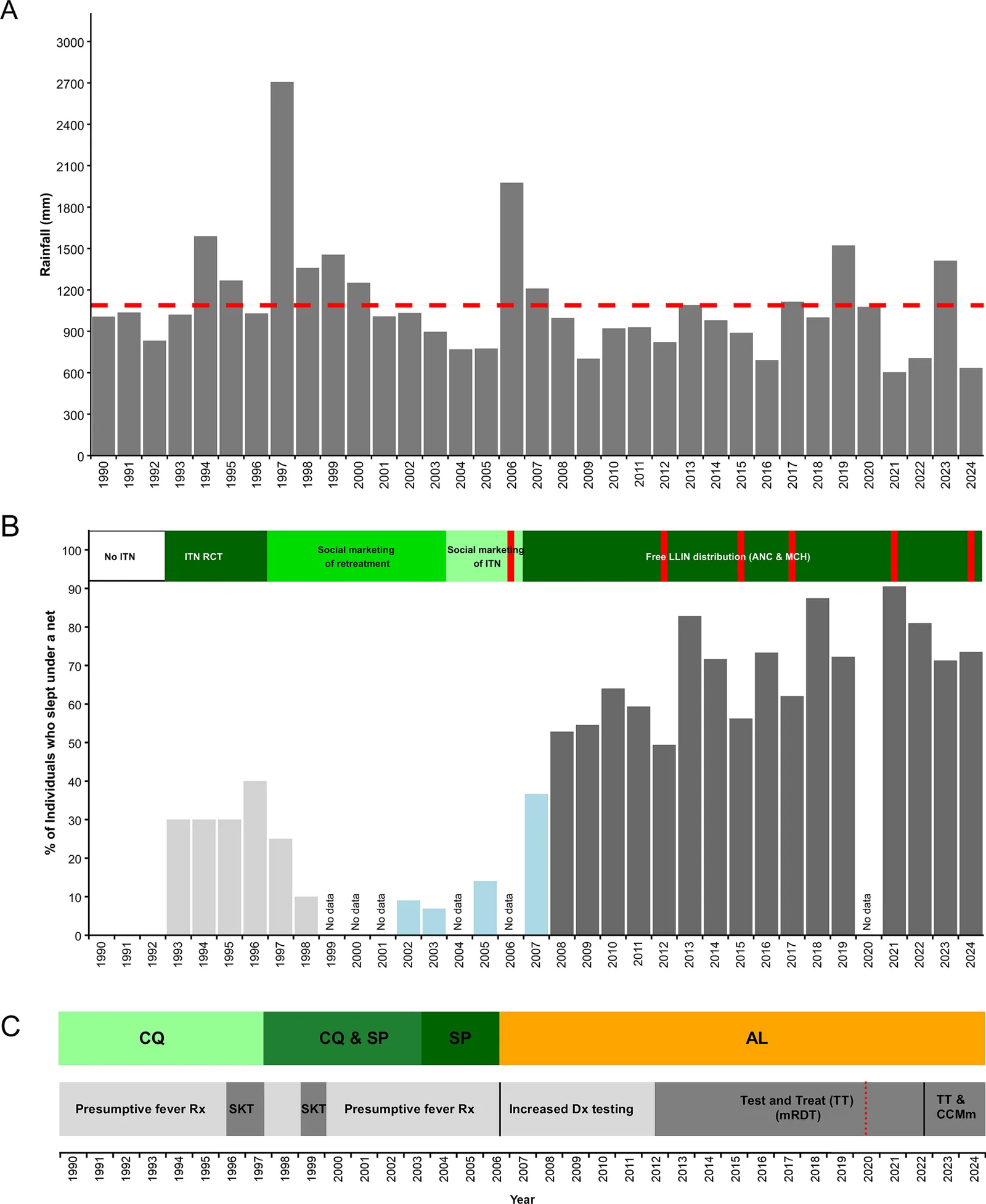
Annual events 1990–2024 related to **A** Rainfall, **B** vector control and **C** case-management (C); summarised across periods in Table 1 and detailed in Additional file 2. **A**: Annual rainfall recorded at the Kilifi Plantation located 2 km south of Kilifi creek. Dotted red line represents the mean annual rainfall over the 35 years of surveillance. **B**: Top bar represents changing delivery approaches for ITN and LLIN since 1993 from the first trial to social marketing, routine subsidised antenatal care (ANC) and maternal child health clinic (MCH) provision, the first mass-free distribution campaign in 2006 (red bar) that heralded free routine ANC and MCH distributions and subsequent mass campaigns shown as red bars. Mid-panel represents the coverage of all age-groups reporting sleeping under a treated net the night before a survey derived from a variety of sources (Supplementary Information 1.4). **C** Summary of treatment policy, guidelines and access. CQ: chloroquine; SP: sulphadoxine-pyrimethamine; AL: artemether-lumefantrine; SKT: Shop Keeper Training programme; Rx: treatment; Dx: diagnosis; TT: test and treat policy introduced in 2006 and revised in 2010 but more completely implemented in 2012 following extensive roll-out of malaria rapid diagnostic tests (mRDT), prior to 2007 most malaria cases were presumptively diagnosed (Dx) and treated (Rx). The first use of community health promoters to Dx (mRDT) and Rx (AL) as part of community case management of malaria (CCMm) began in 2023. Red dotted line in 2020 represents COVID pandemic

Since 1990, Kilifi has undergone multiple changes in malaria treatment, vector bionomics and vector control policies. Figure 1 summarises the contextual factors between 1990 and 2024 related to rainfall, vector control and malaria case-management, more detailed descriptions are available in Additional file 2 and five significant context periods are summarised in Table 1.

**Table 1:** Five major periods defined based on insecticide treated nets/long-lasting insecticidal nets (ITN/LLIN), vector dynamics, drug resistance, case-management policy and rainfall since 1990 (see Additional Files 2 and 3 for detailed context descriptions)

Period	Major threats and intervention milestones
1990–1996	Failing chloroquine (CQ) available through the retail sector and widely used presumptively to treat fevers; gradual ITN introduction as part of a trial in the northern KHDSS; vectors were predominantly *An.gambiae* s.s. biting indoors
1997–2005	CQ replaced by Sulphadoxine-Pyrimethamine (SP) for first line treatment, available in the retail sector for presumptive treatment, but emerging rapid treatment failures across this period; ITN distributions and net re-treatments using social marketing resulting in low coverage; behavioral and species changes of dominant vectors favoring *An.funestus* s.l. and *An.arabiensis*; dramatic climate event (El Niño, 1997) resulting in heavy flooding
2006–2011	SP abandoned, and artemether-lumefantrine (AL) policy introduced only to formal health sector with a complicated testing policy not fully implemented; the first mass distribution LLINs in 2006 and a policy change to provide free routine LLIN access through maternal and child health/antenatal care clinics leading to increased coverage; continued dominant vector composition changes, outdoor/early biting and a low human blood index; relatively low rainfall/droughts (except 2006)
2012–2019	Expanded Test & Treat (TT) policy with increased distributions of malaria rapid diagnostic tests; three mass LLIN campaign distributions (2012, 2015, 2017) with sustained high LLIN coverage and pyrethroid sensitivity; unusually heavy rain 2019
2020–2024	Evidence of high AL and TT health worker treatment policy compliance; initiation of community case management of malaria closer to homes; two mass LLIN campaigns (2021, 2024) with sustained high LLIN use

Prevalence and disease incidence have been assessed across these contextual periods using a Poisson regression model with 1990–1996 as the reference. Changes in median age distributions of malaria admissions and phenotypes were evaluated using the Kruskal–Wallis rank-sum test. Malaria case fatality rate (CFR) was calculated as the proportion of deaths among malaria patients who neither self-discharged nor were transferred to other health facilities. Differences in CFR across the five contextual periods were assessed using a binomial regression model, with 1990–1996 as the reference. All statistical analyses were conducted with R (version 4.4.2).

## Results

Between January 1990 and December 2024, 141,384 paediatric admissions were observed. Exclusions included 25,121 (17.8%) neonates, nine (0.01%) children aged > 14 years, 218 (0.2%) with missing age, 3,553 (2.5%) with missing address, and 43,318 (31%) who were not resident of rural KHDSS. Of the 69,165 admissions included in the analysis, 2,654 (3.8%) were missing blood slide results.

### Community parasite prevalence

**Fig. 2:**
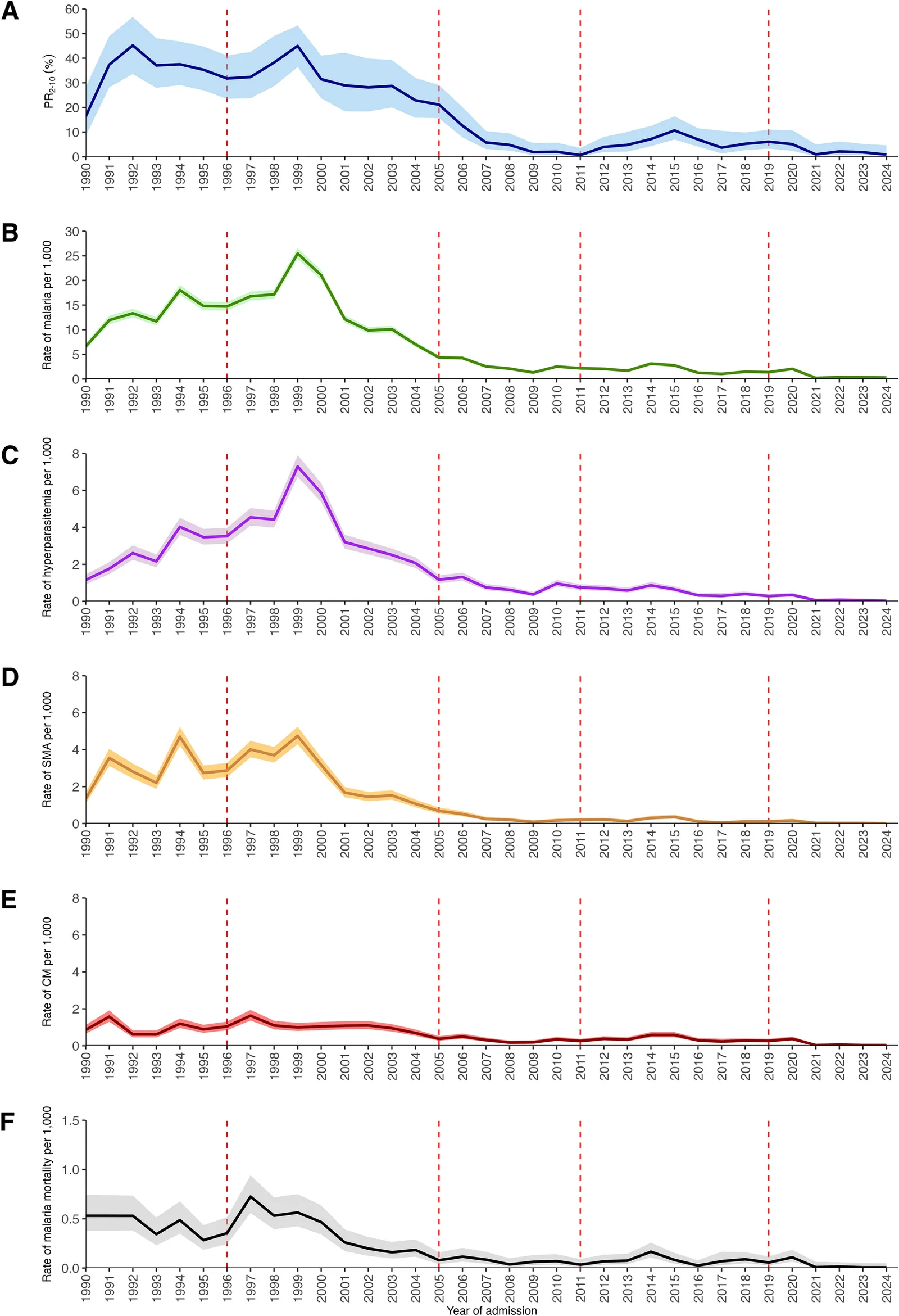
Changing patterns 1990–2024 in background parasite prevalence (**A**), admission rates per 1000 children p.a. of all-cause malaria (**B**), and malaria phenotypes (hyper-parasitaemia (**C**), severe malaria anaemia (**D**), cerebral malaria (**E**) and in-hospital mortality due to malaria (**F**). Dashed red horizontal lines show the five-significant context periods described in Table 1. Among malaria admissions, parasite density was missing in 73 (0.3%) cases, haemoglobin in 331 (1.6%) and an assessment of consciousness was missing in 193 (0.9%) cases

There were 4,443 (6.7%) admissions aged 1 month-14 years from the rural KHDSS that were admitted for trauma, elective surgery, bites and neoplasms and had a blood slide taken. These data were used to estimate the annual age-standardised parasite prevalence, representing community malaria transmission intensity. Prevalence increased between 1990 and 1992, then remained stable at 35–45% through to 1999. From 2000 onwards, parasite prevalence declined steadily reaching an average of 5% (95% CI 3, 6; p < 0.001) between 2006 and 2011. It remained below 10% thereafter, with a modest resurgence observed between 2012 and 2015. In the most recent five-year period (2020–2024), community prevalence averaged 2% (95% CI 1, 4; p < 0.001) indicating a further reduction in exposure to infection among children across the rural KHDSS (Fig. 2A; Table 2).

**Table 2:** Summary statistics on background parasite prevalence, all-cause malaria, malaria phenotypes (hyper-parasitaemia, severe malaria anaemia (SMA), and cerebral malaria (CM)) and malaria specific mortality across five significant context periods described in Table 1

	1990–1996	1997–2005	2006–2011	2012–2019	2020–2024
Trauma/surgical admission age-corrected (2–10 years) SPR; (CI), [N]; p-value^‡^	35% (31, 39) [646]	34% (30, 37) [925]; p = 0.14	5% (3, 6) [951]; p < 0.001	7% (5, 9) [1,237]; p < 0.001	2% (1, 4) [684]; p < 0.001
Acute admissions with a diagnosis of malaria at discharge, % (CI) (n/N)	43.9% (43.2, 44.8) [6,452/14,679]	41.7% (41.1, 42.3) [10,686/25,604]	15.7% (15.0, 16.4) [1,623/10,323]	20.6% (19.7, 21.5) [1,735/8,435]	8.1% (7.4, 8.8) [432/5,357]
Malaria admission rate per 1000 p.a. (CI), [N]; p-value^¶^	13.2‰ (12.8, 13.5) [6,452]	13.3‰ (13.1, 13.6) [10,686]; p = 0.37	2.47‰ (2.35, 2.59) [1,623]; p < 0.001	1.89‰ (1.80, 1.98) [1,735]; p < 0.001	0.65‰ (0.59, 0.72) [432]; p < 0.001
Median age in months (IQR) of malaria admissions [N]	19 (9, 35) [6,452]	23 (12, 39) [10,686]	35 (23, 54) [1,623]	48 (28, 78) [1,735]	43 (26, 77) [432]
Hyper-parasitaemia malaria admission rate per 1000 p.a., (CI), [N]; p-value^¶^	2.72‰ (2.58, 2.87) [1,334]	3.65‰ (3.52, 3.79) [2,925]; p < 0.001	0.79‰ (0.72, 0.86) [517]; p < 0.001	0.52‰ (0.48, 0.57) [477]; p < 0.001	0.11‰ (0.09, 0.14) [73]; p < 0.001
Median age in months (IQR) of hyper-parasitaemia admissions [N]	23 (11, 39) [1,334]	26 (14, 42) [2,925]	36 (25, 54) [517]	49 (31, 80) [477]	44 (29,69) [73]
SMA admission Rate per 1000 p.a. (CI) [N]; p-value^¶^	2.91‰ (2.76, 3.06) [1,425]	2.34‰ (2.24, 2.45) [1,877]; p < 0.001	0.23‰ (0.20, 0.27) [152]; p < 0.001	0.18‰ (0.15, 0.21) [163]; p < 0.001	0.05‰ (0.03, 0.07) [31]; p < 0.001
Median age in months (IQR) of SMA admissions [N]	15 (7, 32) [1,425]	17 (9, 31) [1,877]	28 (19, 42) [152]	34 (18, 58) [163]	35 (23, 50) [31]
CM admission Rate per 1000 p.a. (CI), [N]; p-value^¶^	0.97‰ (0.89, 1.06) [476]	0.98‰ (0.91, 1.05) [781]; p = 0.94	0.30‰ (0.26, 0.34) [195]; p < 0.001	0.38‰ (0.34, 0.42) [347]; p < 0.001	0.11‰ (0.09, 0.14) [71]; p < 0.001
Median age in months (IQR) of CM admissions [N]	27 (15, 45) [476]	27 (15, 40) [781]	38 (27, 58) [195]	57 (35, 82) [347]	52 (32, 94) [71]
Ratio CM/SMA	0.33	0.42	1.30	2.17	2.20
In-patient malaria case-fatality rate (CRF), % (CI) (n/N); p-value^‡^	3.29% (2.88, 3.75) [212/6,452]	2.52% (2.24, 2.84) [269/10,668]; p = 0.003	2.72% (2.03, 3.63) [44/1,619]; p = 0.24	4.25% (3.39, 5.32) [73/1,717]; p = 0.05	4.41% (2.83, 6.81) [19/431]; p = 0.21
Malaria mortality rate per 1000 p.a. (CI), [N]; p-value^¶^	0.43‰ (0.38, 0.49) [212]	0.34‰ (0.30, 0.38) [269]; p = 0.006	0.07‰ (0.05, 0.09) [44]; p < 0.001	0.08‰ (0.06, 0.10) [73]; p < 0.001	0.03‰ (0.02, 0.05) [19]; p < 0.001
Median age in months (IQR) of malaria mortality [N]	21 (9, 36) [212]	24 (9, 38) [269]	36 (24, 51) [44]	53 (33, 77) [73]	42 (26, 94) [19]

^**‡**^The differences between proportions across the five major context periods were assessed using a binomial regression with the 1990–1996 as the reference period

^¶^The differences between incidence rate across the five major context periods were assessed using a Poisson regression model, with the population estimates included as an offset and 1990–1996 as the reference period

### Hospitalised malaria

During the 35-year surveillance period, there were 20,928 admissions with a malaria diagnosis, accounting for 34% of admissions; 17,755 (85%) had malaria as the sole diagnosis, 1,950 (9%) had malaria as the primary diagnosis with a secondary condition, and in 1,223 (6%) malaria was a secondary diagnosis. The annual rate of malaria hospitalisation among children aged 1 month-14 years increased sharply during the 1990s. In 1990, the malaria admission rate was 6.6 per 1,000 children p.a. (95% CI 6.0, 7.2) and malaria accounted for 27% of admissions. By 1999, malaria hospitalisation rates had risen to a peak of 25.5 per 1,000 children p.a. (95% CI 24.4, 26.6), representing 53% of admissions (Fig. 2B). From 2000 onwards, rates began to decline steadily: 21.1 per 1,000 children p.a. (95% CI 20.0, 22.0) in 2000 to 1.32 per 1,000 children p.a. (95% CI 1.12, 1.55) by 2009 (Fig. 2B).

Across the period of changing intervention (Table 1), malaria accounted for 43.9% (95% CI 43.2, 44.8) of paediatric admissions in1990-1996, and 41.7% (95% CI 41.1, 42.3) in 1997–2005. Between 2012 and 2019, malaria hospitalisation rates averaged 1.89 per 1,000 children p.a. (95% CI 1.80, 1.98; p < 0.001) representing only 20.6% of all admissions. The period from 2020 was characterised by a further decline in malaria hospitalisation with an average across the period of 0.65 per 1,000 children p.a. (95% CI 0.59, 0.72; p < 0.001), with only 23–53 malaria admissions between 2021 and 2024 accounting for less than 5% of all admissions (Fig. 2B; Table 2).

### Severe malaria phenotypes

Trends in the rates of annual hospitalised malaria admissions with hyper-parasitaemia, severe malaria anaemia and cerebral malaria are shown in Fig. 2C-2E. Broadly, the rise and fall in rates of hyper-parasitaemia and SMA were comparable to those described for malaria admission rates (Fig. 2B). For example, SMA admission rates at the peak of malaria case burdens in 1999 was 4.74 per 1,000 children p.a. (95% CI 4.29, 5.23), compared to the lowest admission period, 2020–2024, where the average SMA hospitalisation rate was 0.05 per 1,000 children p.a. (95% CI 0.03, 0.07; p < 0.001) (Fig. 2D; Table 2). Although the absolute incidence of cerebral malaria declined over time, the ratio of CM to SMA increased over time, from 0.3 before 2006, increased to 1.3 in 2006–2011 and increased further to 2.2 between 2012 and 2024 (Table 2).

### Hospitalised malaria mortality

Among children admitted with a malaria diagnosis, 617 (2.9%) died before discharge. CFR varied modestly over time, from 3.3% (95% CI 2.9, 3.8) in 1990–1996 to 2.5% (95% CI 2.2, 2.8; p = 0.003) in 1997–2005. During the lowest transmission periods, the CFR was 2.7% (95% CI 2.0, 3.6; p = 0.24) in 2006–2011 and 4.4% (95% CI 2.8, 6.8; p = 0.21) in 2020–2024. The malaria-specific hospitalised mortality rate was 0.43 per 1,000 children p.a. (95% CI 0.38, 0.49) during 1990–1996 and declined steadily thereafter, reaching 0.03 per 1,000 p.a. (95% CI 0.02, 0.05; p < 0.001) by 2020–2024 (Table 2; Fig. 2F).

### Changing age patterns

**Fig. 3:**
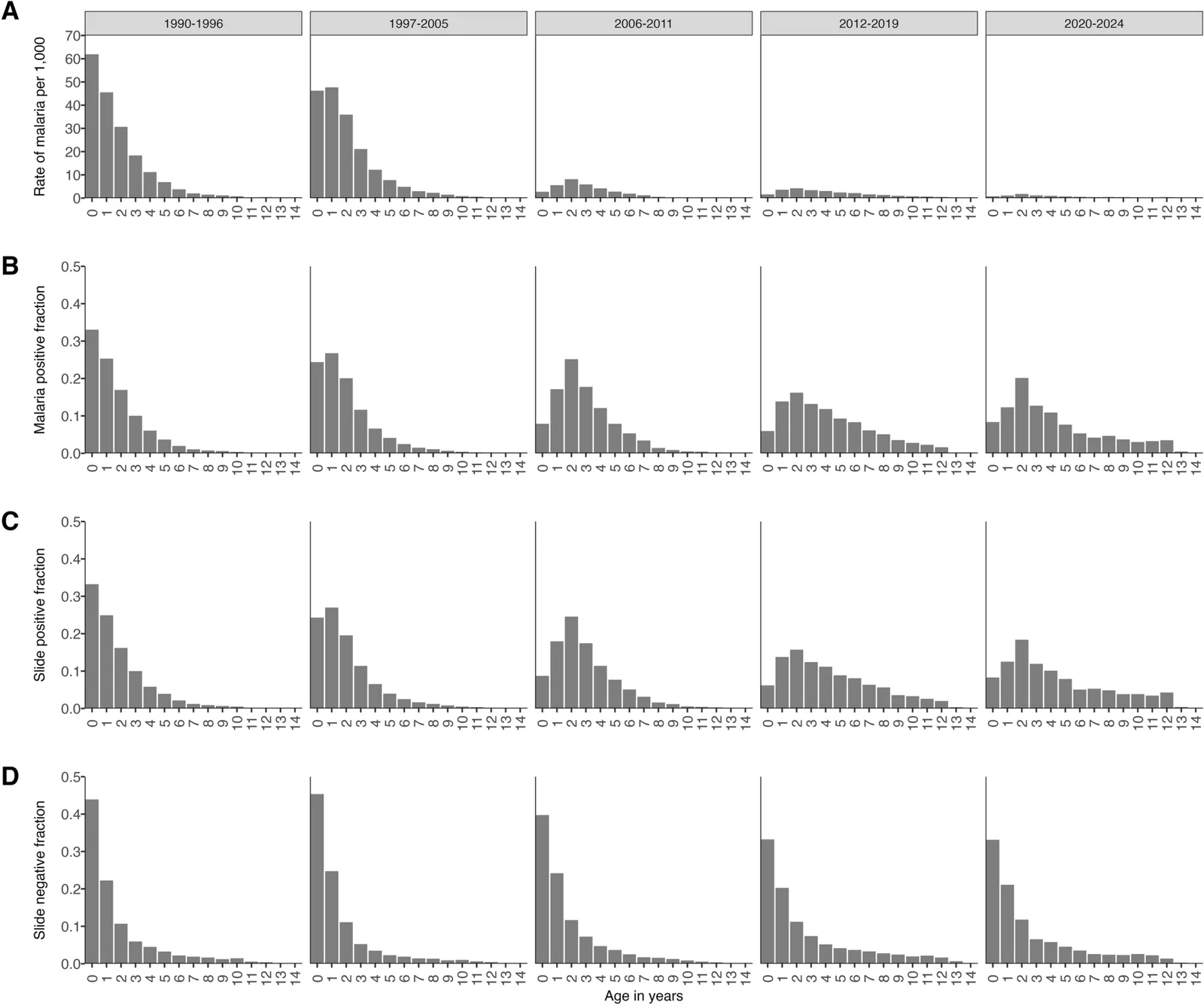
Age patterns of rates of malaria admission (**A**), fractions of malaria admission (**B**), slide-positive (**C**) and slide-negative (**D**) over five-time intervals among children age 1 m-14 years at Kilifi County Hospital, 1990–2024

As transmission intensity and rates of malaria hospitalisation declined, the median age of malaria hospitalisation increased (Fig. 3A and 3B; Table 2), rising from 19 months (IQR: 12, 39) between 1990–1996 to 48 months (IQR: 28, 78) between 2012–2019. Similar changes to the age-specific rates were observed across the three severe malaria phenotypes, with the median age increasing as transmission intensity declined, although the absolute age varied by phenotype (Table 2). The age distribution of malaria shifted over time, with older children increasingly represented in the rate of malaria hospitalisation (Fig. 3A) and proportional malaria admissions (Fig. 3B), however this shift did not result in an overall increase in rate of malaria hospitalisation (Fig. 2B). The age pattern observed for children with a discharge diagnosis of malaria (Fig. 3B) was similar to those who were reported as slide positive on admission, regardless of diagnosis (Fig. 3C) but slide negative admissions showed a more stable age pattern across all time periods (Fig. 3D).

## Discussion

This analysis characterises the long-term trends in the risk and age patterns of severe hospitalised malaria in a rural population on the Kenyan coast over a 35-year period. There has been a 94% reduction in community malaria prevalence since the 1990s (Fig. 2A). Consequently, children born within the KHDSS today face a considerably lower risk of malaria infection compared to those born during the 1990s, accompanied by a dramatic decline in malaria hospitalisation from the same community (Fig. 2B). Since 1990, the annual burden of hospitalised paediatric malaria at KCH has fallen from thousands of cases to fewer than 100 per year. Malaria accounted for 43% of paediatric admissions 1990–2005, and since 2021 represents less than 5%. Severe malaria anaemia and hyper-parasitaemia admission rates followed a similar temporal pattern (Figs. 2C, D). Although cerebral malaria now represents a larger proportion of severe cases, the absolute number of cases remain very low (Table 2). In-hospital malaria mortality among children from the KHDSS has declined by 93% since 1990, from 0.43 per 1,000 p.a. (1990–1996) to 0.03 per 1,000 p.a. (2020–2024), and by 63% between 2012–2019 and 2020–2024 (Table 2; Fig. 2F).

As transmission intensity and hospitalisation rates declined, the median age of malaria admissions and all slide-positive admissions increased, while the age pattern of children admitted without malaria infection remained constant over the surveillance period (Fig. 3). It has been suggested that declining parasite exposure may shift the peak age of severe disease to older children, potentially offsetting overall reductions in risk during childhood [16, 17], and that increased vulnerability of older children to cerebral malaria may offset reductions in mortality. This pattern was not observed in the present analysis. As transmission intensity declined, older children had a relatively higher risk of hospitalisation, but the absolute numbers remained low, and this increase did not result in rise or stagnation in cumulative risks (Figs. 2, 3; Table 2). However, adult ward surveillance was not maintained throughout the 35-year observation period, and we cannot rule out an increase in adult admissions in later years. As transmission declines and age-specific clinical immunity changes there is a need to extend surveillance beyond paediatric populations.

### Plausible contributions to the changing malaria epidemiology

The drivers of changing parasite exposure and disease progression within the KHDSS over the past 35 years are multi-layered and complex (Additional file 2). There are several plausible congruences between policy implementation and changes in infection prevalence and disease incidence. In the late 1990s, two coinciding factors may have accelerated the changing transmission intensity. First, the widespread and easily accessible use of long-half-life, “prophylactic” treatments for fever, sulphadoxine-pyrimethamine (SP). Second, a rapid change in vector composition and behaviour associated with increasing ITN use. This period saw a transition from the historically dominant, anthropophilic *Anopheles gambiae* s.s to more opportunistic vectors (*An. arabiensis* and *An. funestus,* accompanied by increased outdoor biting and a change from almost universal human blood meals in the 1990s to < 15% by 2010 [18] (Additional file 2 section 2.3).

Pyrethroid sensitivity bioassay conducted between 1994 and 2014 continued to show high vector mortality to pyrethroids (Additional file 2 section 2.4). Importantly, studies within the KHDSS between 2008 and 2019 consistently showed that long-lasting insecticide treated nets (LLIN) use among children was associated with a 55% reduction in community-based infection prevalence (adjusted OR = 0.45; 95% CI 0.36 to 0.57) [19]; a 32% reduction in out-patient malaria slide positivity (crude OR = 0.68; 95% CI 0.64, 0.73) [20]; and a 33% reduction in malaria admission (HR = 0.67; 95% CI 0.52, 0.85) [21].

Treatment of uncomplicated malaria with chloroquine until 1996 may have contributed to rising hospitalisations, and its replacement with SP, a drug with already reduced efficacy (Additional file 2 section 2.5), did not change the rise in malaria admissions (1997–2005). During the early phases of policy change to artemether/lumefantrine (AL) (2006–2011), treatment access was largely restricted to formal health care providers and adherence to guidelines was sub-optimal (Additional file 2 section 26). Following the decentralisation of health services in 2010, there was an increase in health facilities within the KHDSS, alongside renewed efforts to improve malaria case-management improving use and compliance with diagnostics (rapid diagnostic tests (RDT)) and treatment (AL) (2012–2019) (Additional file 2 section 2.6). Since 2023, there has been an expansion of community case management with community health promoters provided with RDTs and AL across the KHDSS (Additional file 2 section  2.7). Evidence from the KHDSS indicates that AL remains an effective first line treatment [22]. Although, the direct effects of prompt, effective treatment on disease progression requiring hospital care are difficult to quantify, it is likely that improved access to effective treatment has contributed to the continued decline in malaria admissions.

Major precipitation anomalies in 1997 associated with El Niño (Additional file 3) coincided with low ITN coverage (Additional file 2 section 2.2) and drug resistance potentially creating a “perfect storm” for malaria transmission. Mean day-time temperatures within the KHDSS have risen by nearly 1 °C since 2003 [23]. However, heavy rains events in 2006, 2019 and 2023 (Additional file 3) and rising temperatures since 2003 were not associated with major increases in malaria hospitalisation. While climate change may affect malaria risk at continental scales, within the KHDSS, climate anomalies appear less influential than sustained coverage and effectiveness of long-lasting insecticide treated nets (LLIN), and improved access to effective first-line treatment.

Broader socioeconomic and infrastructural changes may have contributed to the observed trends. However, the KHDSS has not systematically collected longitudinal data on household wealth, mobile phone ownership and use of mobile financing, maternal knowledge of malaria, education level or housing types. At the national level, these factors have improved substantially since 1990 [24] and it is likely similar improvements have occurred within the KHDSS.

The question therefore is whether the local patterns of malaria since 2006 represent a “new normal”, with infection prevalence below *circa* 5% and malaria hospitalisation rates < 5 per 1000 children p.a. In the absence of a sterilising or transmission blocking vaccine, it is unlikely that parasite transmission will be interrupted within the KHDSS in the foreseeable future. A more realistic goal is to prevent malaria deaths. Despite emerging threats from parasite and vector resistance (Additional file 2 Sect. 2.4 and 2.5), this can be achieved through expanded and prompt access to effective treatment, combined with a sustained supply of LLIN. Any adjustments to diagnostics, treatment and vector control must be guided by continuous surveillance of parasite and vector resistance, vector species compositions, efficacy testing and epidemiological data. Without surveillance at local levels, policy adaptions will lack empirical evidence and are likely to be delayed.

## Conclusion

There has been a sustained, dramatic reduction in paediatric malaria hospitalisation over the past 35 years. During the 1990s, malaria accounted for more than 40% of all paediatric admissions, whereas today malaria requiring emergency care is relatively rare. As hospitalisation incidence has declined, the clinical epidemiology has shifted: children admitted now are older and more likely to have cerebral malaria than severe malaria anaemia. Nevertheless, the overall incidence of hospitalisation and cerebral malaria in older children continues to decline. Despite concerns that progress might stagnate, this has not been observed in the Kilifi study area. Two core interventions, LLIN and prompt access to AL, have likely been pivotal in reducing severe malaria and associated deaths in this setting. However, if reductions in ODA are not compensated for by increased domestic investment, this will lead to reduced coverage of effective malaria interventions and might reverse the substantial gains achieved in malaria burden reduction.

## Supplementary Information

Additional file 1.

Additional file 2.

Additional file 3.

## Data Availability

These data are available through a formal requesting process to the KEMRI-Wellcome Trust Institutional Data Access/Ethics Committee. Correspondence and requests for materials should be addressed to the KEMRI Wellcome Data Governance Committee (dgc@kemri-wellcome.org).

## Abbreviations

AL: Artemether-lumefantrine
ANC: Antenatal care
BCS: Blantyre coma score
CFR: Case fatality rate
CQ: Chloroquine
CI: Confidence interval
CM: Cerebral malaria
Dx: Diagnosis
CCMm: Community case management of malaria
HR: Hazard ratio
ICD: International classification of diseases
IQR: Interquartile range
ITN: Insecticide-treated bed net
KCH: Kilifi county hospital
KEMRI: Kenya medical research institute
KHDSS: Kilifi health and demographic surveillance system
KWTRP: KEMRI–Wellcome trust research programme
LLIN: Long-lasting insecticide treated nets
MCH: Maternal child health clinic
MoH: Ministry of health
mRDT: Malaria rapid diagnostic tests
ODA: Overseas development assistance
OR: Odds ratio
RBM: Roll back malaria
RDT: Rapid diagnostic test
Rx: Treated
SMA: Severe malaria anaemia
SP: Sulphadoxine-pyrimethamine
TT: Test and treat policy
WHO: World Health Organization
