# Supplementary material for: Decline to near-zero malaria hospitalisation over 35 years on the Kenyan Coast
Source: Malar J. 2026 Aug 7;25:399. doi: 10.1186/s12936-026-06081-x (PMC13732978; doi:10.1186/s12936-026-06081-x)
Supplement: Supplementary file 1 [file 12936_2026_6081_MOESM1_ESM.docx]

## **Additional File 1: Kilifi study area and population**

Studies on severe malaria and its epidemiology in the area surrounding Kilifi hospital (KCH) began during the period 1989-1993 with Wellcome Trust funding to the University of Oxford in collaboration with the Kenya Medical Research Institute (KEMRI) including descriptions of the clinical spectrum of severe paediatric malaria [1], severe anaemia [2], the validity of Verbal Autopsies in the attribution of paediatric causes of death [3], case-control studies on severe disease progression [4,5] and parallel, allied studies related to entomological risk factors [6] and human behaviour [7]. The research platform enabled linkage of hospitalised events to locations and sub-locations surrounding KCH. Demographic surveillance for the entire study area was established in 2002 [8] enabling linkage to resident household members living in 186 enumeration zones (EZ) across 15 locations (Figure 1). The rural population in 2003 was 183,509 and grew to 286,078 by 2024. In and out migration from the KHDSS is approximately 8.8% [8]. Continuous community engagement activities have been maintained for over 20 years to explain the purposes of the research to the community [9,10].

Most residents of the KHDSS belong to the Mijikenda group while other ethnicities have settled in the area from Central and Western Kenya with a notable mixture of different groups in Kilifi Township. Among the rural population, agriculture forms the basis of most household activity. Adult male household members who work outside the KHDSS provide an important source of financial remittance for the community. Kilifi Township, and the area immediately south of the creek connected by a bridge (Mnarani), has grown significantly since 1990 and comprise traders, artisans, county government and public service administrators, police, hospital personnel and those employed in the transport and tourist industries. Kilifi township includes a hospital maintenance school that provides training for students from across Kenya, the KEMRI research campus grew significantly from 1990 and now provides employment for over 700 staff, Pwani University was established in 2007 and provides courses for *circa* 8000 students all housed within the township area, there are four international hotels and the Kilifi Sisal Plantation is located south of Kilifi Creek. These centres provide employment for people from across Kenya in the township area. This area (Figure 1; dark grey) is characterised by substantial population mobility and a high proportion of transient residents. Because these dynamics complicate the attribution of hospitalisations to a well-defined, stable catchment population, admissions from this area were excluded.

**Figure 1:** Location of Kilifi Health and Demographic Surveillance Site showing location of Kilifi County Hospital (KCH), the urban extents excluded from analysis (dark grey), households (light grey dots) and the main Malindi-Mombasa road (red). Grey boundaries show administrative locations.

**References**

1. Marsh K, Forster D, Waruiru C, Mwangi I, Winstanley M, Marsh V, et al. Indicators of life-threatening malaria in African children. N Engl J Med. 1995;332:1399–404. https://doi.org/10.1056/NEJM199505253322102

2. Newton CR, Warn PA, Winstanley PA, Peshu N, Snow RW, Pasvol G, et al. Severe anaemia in children living in a malaria endemic area of Kenya. Trop Med Int Health. 1997;2:165–78. https://doi.org/10.1046/j.1365-3156.1997.d01-238.x

3. Snow RW, Armstrong JR, Forster D, Winstanley MT, Marsh VM, Newton CR, et al. Childhood deaths in Africa: uses and limitations of verbal autopsies. Lancet. 1992;340:351–5. https://doi.org/10.1016/0140-6736(92)91414-4

4. Snow RW, Schellenberg JR, Peshu N, Forster D, Newton CR, Winstanley PA, et al. Periodicity and space-time clustering of severe childhood malaria on the coast of Kenya. Trans R Soc Trop Med Hyg. 1993;87:386–90. https://doi.org/10.1016/0035-9203(93)90007-d

5. Snow RW, Peshu N, Forster D, Bomu G, Mitsanze E, Ngumbao E, et al. Environmental and entomological risk factors for the development of clinical malaria among children on the Kenyan coast. Trans R Soc Trop Med Hyg. 1998;92:381–5. https://doi.org/10.1016/s0035-9203(98)91056-2

6. Mbogo CN, Snow RW, Khamala CP, Kabiru EW, Ouma JH, Githure JI, et al. Relationships between Plasmodium falciparum transmission by vector populations and the incidence of severe disease at nine sites on the Kenyan coast. Am J Trop Med Hyg. 1995;52:201–6. https://doi.org/10.4269/ajtmh.1995.52.201

7. Mwenesi HA, Harpham T, Marsh K, Snow RW. Perceptions of symptoms of severe childhood malaria among Mijikenda and Luo residents of coastal Kenya. J Biosoc Sci. 1995;27:235–44. https://doi.org/10.1017/s0021932000022720

8. Scott JAG, Bauni E, Moisi JC, Ojal J, Gatakaa H, Nyundo C, et al. Profile: The Kilifi Health and Demographic Surveillance System (KHDSS). Int J Epidemiol. 2012;41:650–7. https://doi.org/10.1093/ije/dys062

9. Marsh V, Kamuya D, Rowa Y, Gikonyo C, Molyneux S. Beginning community engagement at a busy biomedical research programme: experiences from the KEMRI CGMRC-Wellcome Trust Research Programme, Kilifi, Kenya. Soc Sci Med. 2008;67:721–33. https://doi.org/10.1016/j.socscimed.2008.02.007

10. Kamuya DM, Marsh V, Kombe FK, Geissler PW, Molyneux SC. Engaging communities to strengthen research ethics in low-income settings: selection and perceptions of members of a network of representatives in coastal Kenya. Dev World Bioeth. 2013;13:10–20. https://doi.org/10.1111/dewb.12014
